# Modeling the Impact of Malaria Chemoprevention with sulphadoxine-pyrimethamine on the spread of *Plasmodium falciparum dhps* A581G mutation

**DOI:** 10.1016/j.idm.2026.03.004

**Published:** 2026-03-14

**Authors:** Nicholaus Mziray, Lucy C. Okell, Samson Kiware, Gina Cuomo-Dannenburg, Neterindwa Ainea, Deus S. Ishengoma, Nkuba Nyerere

**Affiliations:** a Ifakara Health Institute, Environmental Health, and Ecological Sciences Department, P.O. Box 78373, Kiko Avenue, Mikocheni, Dar es Salaam, the United Republic of Tanzania; b Department of Mathematics and Statistics, Sokoine University of Agriculture, P.O.Box 3000, Morogoro, the United Republic of Tanzania; c MRC Centre for Global Infectious Disease Analysis, Department of Infectious Disease Epidemiology, Imperial College London, UK; d The Nelson Mandela, African Institution of Science and Technology, The School of Life Science and Bioengineering (LISBE), P. O. Box 447, Tengeru, Arusha, the United Republic of Tanzania; e National Institute for Medical Research, Dar es Salaam, the United Republic of Tanzania

**Keywords:** Sulphadoxine-pyrimethamine, Malaria, Malaria chemoprevention, Resistant mutations, Mathematical modeling

## Abstract

Sulfadoxine-pyrimethamine (SP) is a key antimalarial used in chemoprevention strategies, including intermittent preventive treatment in pregnancy (IPTp); intermittent preventive treatment in infants (IPTi); seasonal malaria chemoprevention (SMC); and mass drug administration (MDA). Malaria chemoprevention using sulfadoxine-pyrimethamine (SP) has shown a positive impact in preventing millions of malaria cases in the setting were it has widely been implemented. However, the spread of SP resistance in *Plasmodium falciparum*, particularly the emergence of the *dhps* A581G mutation which confers fully resistance to SP, threatens its long-term effectiveness. This study uses a mathematical modeling approach to evaluate how different chemoprevention strategies could influence the spread of SP-resistant genotypes in settings where *dhps* K540E mutation is already near fixation (highly prevalent). We developed a deterministic age-structured compartmental model incorporating both human and mosquito populations. The study simulates malaria transmission dynamics and the competition between *dhps* K540E and resistant *dhps* A581G parasite strains under varying chemoprevention coverage levels and treatment cycles. Simulations were carried out considering the settings where *dhps* K540E is most prevalent and is already near fixation, while *dhps* A581G is rising in prevalence. Two intervention scenarios were examined: three monthly chemoprevention cycles and five monthly cycles per transmission season, with coverage levels varrying from 0% to 80%. Model simulations demonstrate that chemoprevention coverage and frequency significantly affect the spread of *dhps* A581G. At 45% chemoprevention coverage, *dhps* A581G increased from 11% to 20.6% (three cycles) to 27.8% (five cycles). ANOVA was performed, followed by a partial eta-squared $\left(\eta_{p}^{2}\right)$ to quantify the size effects of coverage levels and the number of treatment cycles while controlling for other variables in the model. Results revealed that both the chemoprevention coverage levels and the number of treatment cycles influence the spread of resistance, with 58% of the spread of resistance $\left(\eta_{P}^{2}=0.58,95\%\text{ CI: }0.57-1.00\right)$ explained by the chemoprevention coverage level, making coverage level the most influential factor affecting the spread of resistance. The number of treatment cycles per year also matters, but its influence on the spread of resistance is relatively smaller, only 9.3% of the spread of resistance $\left(\eta_{P}^{2}=0.093,95\%\text{ CI: }0.09-1.00\right)$ is explained by the number of treatment cycles. The interaction between chemoprevention coverage levels and the treatment cycles influences the spread of resistance by 9.0%, $\left(\eta_{P}^{2}=0.090,95\%\text{ CI: }0.08-1.00\right)$. Our findings highlight a trade-off between immediate public health gains from high chemoprevention coverage and the long-term risk of accelerating SP resistance. While chemoprevention using SP influences the spread of the *dhps* A581G mutation, careful policy planning, decisions, and ongoing molecular surveillance are essential to optimize the benefits of chemoprevention while minimizing the risk of the spread of the *dhps* A581G mutation.

## Highlights

• Impact of malaria chemoprevention using SP on the spread of *Plasmodium falciparum* A581G mutations on the *dhps*gene is studied.
• Reproduction numbers for wild-type and resistant mutations are calculated using the next-generation matrix.
• Effect of chemoprevention coverage and number of treatment cycles on the spread of resistant mutations are investigated.
• Chemoprevention coverage and number of treatment cycles both influence spread of the resistant mutation.

## 1 Introduction

Malaria remains a major public health challenge in many parts of sub-Saharan Africa causing significant morbidity and mortality in children (WHO, 2024) despite control efforts in place. In 2023, malaria cases worldwide was estimated at 263 million, where sub-Saharan Africa accounted for an estimated 94% of all cases worldwide, the number of malaria deaths was estimated at 597, 000, with sub-Saharan Africa accounting for 95% of all malaria deaths worldwide (WHO, 2024).

Despite significant progress in control and prevention over the past two decades, malaria transmission remains high due to different challenges such as insecticide resistance, climate change, low coverage of interventions, humanitarian emergencies, social-cultural hindrance, the spread of *Anopheles stephensi*, and the emergence and the spread of antimalarial drug resistance (WHO, 2024). To mitigate morbidity and mortality, the World Health Organisation (WHO) recommends several chemoprevention strategies, including the intermittent preventive treatments during pregnancy (IPTp), intermittent preventive treatment in infants (IPTi) currently known as perennial malaria chemoprevention (PMC), seasonal malaria chemoprevention (SMC) in the areas with highly seasonal malaria transmission where more than 60% of malaria cases occur within four consecutive months, and mass drug administration (MDA) (WHO, 2023).

Sulphadoxine-pyrimethamine is central to these preventive approaches due to its long elimination half-life, affordability, and ease of administration (Masserey, Braunack-Mayer, et al., 2024). Malaria chemoprevention strategies such as IPTp and IPTi/PMC, which have been widely implemented using SP, have shown to be effective in preventing malaria cases in eligible groups (Ahadzie-Soglie et al., 2022; Bouyou-Akotet et al., 2016; Matambisso et al., 2024; Walker et al., 2017).

However, resistance to SP, caused by mutations in the *Plasmodium falciparum dihydrofolate reductase (dhfr)* and *dihydropteroate synthase (dhps)* genes, threatens its long-term efficacy. Of particular concern is the A581G mutation in *dhps*, which, when combined with the K540E mutation, is associated with reduced protective efficacy of SP-based chemoprevention (Bwire et al., 2020; Triglia et al., 1997). The rise in the prevalence of the *dhps* A581G mutation poses a critical threat to the sustainability of SP-based chemoprevention strategies.

Mathematical models for the transmission dynamics of antimalarial drug resistance serve as a useful tool to understand different factors influencing the spread of drug resistance, and used to guide the design of rational strategies for the control of the spread of drug resistant parasites.

Previous modeling studies have been used to explore various aspects of antimalarial drug resistance. Okosun and Makinde (2011) derived and analyzed a deterministic model for the transmission of malaria including the class of individuals with drug resistance and treatment measures. Their study found out that effective control of the proportion individuals with drug resistance has a positive impact in reducing the spread of the disease. A study by Brock et al. (2017) used a deterministic compartmental model to quantify the impact of antimalarial medicine quality on the transmission of SP resistance. Results from their study suggest that an increase in poor-quality antimalarial use predicts an increase in the transmission of resistance. Other modeling studies have shown that high coverage and frequent chemopreventive doses can accelerate the selection of resistant strains, (Manore et al., 2019; Masserey, Lee, et al., 2024; Teboh-Ewungkem et al., 2015), and a non modeling study Maiga et al. (2016) in Mali also reported the increase of quintuple mutant genotype among the children receiving chemoprevention from 1.6% to 7.1% after chemoprevention. A study by Mousa et al. (2025) also used a mathematical approach to estimate the duration of SP protection against parasites with different *dhps* genotypes and showed that the duration of SP protection against parasites with fewer mutations in the *dhps* gene was higher compared to parasites with additional mutations in the *dhps* gene.

However, the interaction between chemoprevention using SP deployment parameters and the spread of *dhps* A581G mutation remains underexplored in many settings especially to those settings where *dhps* K540E mutation is most prevalent and already near fixation. This study uses a mathematical modeling framework to assess how variations in chemoprevention coverage and treatment frequency impact the spread of *dhps* A581G mutation in to settings where *dhps* K540E mutation is most prevalent and already near fixation.

We aim to explore the trade-offs of the benefits of chemoprevention while minimizing the risk of the spread of the *dhps* A581G mutation and to inform policy and decisions.

The rest of this work is organized as follows: Section 2 presents material and methods, giving details on model description and formulation and underlying assumptions. Section 3 provided details on model analysis by giving analysis of the model. Section 4 provides details on model simulations and model parameters and numerical results. Section 5 presents discussion of the results. Finally, Section 6 provides a conclusion with a summary of the key findings, policy implications, and the direction for future research.

## 2 Material and methods

### 2.1 Model description and formulation

An age-structured deterministic mathematical model for the transmission dynamics of malaria in human and mosquito subpopulations is formulated in this section. In this model, we consider malaria chemoprevention using SP, administered to children under five years of age. For human population dynamics, therefore, the population is divided into ten compartments. These are further subdivided into five compartments each to account for the individuals who have not taken chemoprevention and those who have taken chemoprevention. For the group without chemoprevention, individuals can be in one of the following states: susceptible *S*_*h*_, individuals who do not show symptoms or untreated infection with wild-type strain *I*_*aw*_, individuals who do not show symptoms or untreated resistant infection *I*_*ar*_, symptomatic and treated wild-type strain infection *I*_*sw*_, and symptomatic and treated resistant infection *I*_*sr*_.

For the group with chemoprevention, individuals can be in one of the following states: susceptible *P*_*h*_, individuals who do not show symptoms or untreated infection with wild-type strain *I*_*apw*_, individuals who do not show symptoms or untreated resistant infection *I*_*apr*_, symptomatic and treated wild-type strain infection *I*_*spw*_, and symptomatic and treated resistant infection *I*_*spr*_. Thus, total human population at any time *t* is given as, *N*_*h*_ = *S*_*h*_ + *P*_*h*_ + *I*_*aw*_ + *I*_*ar*_ + *I*_*sw*_ + *I*_*sr*_ + *I*_*apw*_ + *I*_*apr*_ + *I*_*spw*_ + *I*_*spr*_ on the other hand, the mosquito population is divided into five compartments namely; Susceptible *S*_*v*_, incubation of either wild-type strain infection *E*_*vw*_ or resistant infection *E*_*vr*_, wild-type strain infected *I*_*vw*_ and resistant infected *I*_*vr*_ mosquitoes. Thus, total mosquito population is given as, *N*_*v*_ = *S*_*v*_ + *E*_*vw*_ + *E*_*vr*_ + *I*_*vw*_ + *I*_*vr*_ We do not consider co-infection of strains in our model; therefore, individuals are infected with either the wild-type or resistant strain, but not both. However, we allow super-infection which ‘knocks out’ the existing strain. For example, a new resistant strain might infect a person who already has a wild-type infection and replace it, and for the individual super-infected with the same strain, develops symptoms for that particular strain and moves into the symptomatic and treated state of the same strain. We further assume a constant population where individuals are born at the rate Λ_*h*_ which equals to the natural death rate for the humans *μ*_*h*_.

Susceptible individuals without chemoprevention become infected with either the wild-type or resistant strain, depending on whether a bite was from a mosquito infected with the wild-type or the resistant strain. The force of infection for the individuals in this group is given as *λ*_*hw*_ which corresponds to wild-type strain and *λ*_*hr*_ for the resistant strain. The newly infected individuals have the probability *ξ* of developing symptoms and becoming symptomatic and move into compartment *I*_*sw*_ or *I*_*sr*_. Individuals who do not develop symptoms move either into compartment *I*_*aw*_ or *I*_*ar*_. We assume that resistant strains circulating in the population have the common SP markers, so symptomatic individuals are treated with the first-line treatment drugs which would perform normally and take shorter time before becoming susceptible at the rate *γ*_*s*_ unlike for the untreated individuals who stay longer in these compartments before becoming susceptible again after clearing the infection at the rate *γ*_*a*_. Individuals who clear infections through either treatment or natural immunity lose all the strains to become susceptible.

Susceptible individuals *P*_*h*_ who have received chemoprevention become infected with either wild-type or resistant strain. However, the force of infection for these individuals is affected by the probability of protection which the chemoprevention drug offers for each strain. The force of infection corresponding to the wild-type strain is given as (1 − *ψ*_*w*_(*t*))*λ*_*hw*_ and for the resistant strain is given as (1 − *ψ*_*r*_(*t*))*λ*_*hr*_, where *ψ*_*w*_ and *ψ*_*r*_ are the probabilities of drug protection against the wild-type and resistant strain respectively. This probability of individual being protected by chemoprevention for each strain is modelled as a time dependent and is assumed to follow a Weibull survival curve with scale parameter *λ*_*i*_ and the shape parameter *w*_*i*_, where *i* = (*w*, *r*). At each time step probability of drug protection against the wild-type strain is given as $\psi_{w}=e^{-}{\left(\frac{t}{\lambda_{w}}\right)}^{w_{w}}$ and for the resistant strain is given as $\psi_{r}=e^{-}{\left(\frac{t}{\lambda_{r}}\right)}^{w_{r}}$. The mean duration of protection against new infection by the drug is assumed to be different for each strain. The mean duration of protection *η*_*i*_ for each strain is determined as: $\eta_{i}=\lambda_{i}\Gamma \left(1+\frac{1}{w_{i}}\right)$, where Γ denotes the gamma function and *i* = (*w*, *r*).

**Fig. 1:**
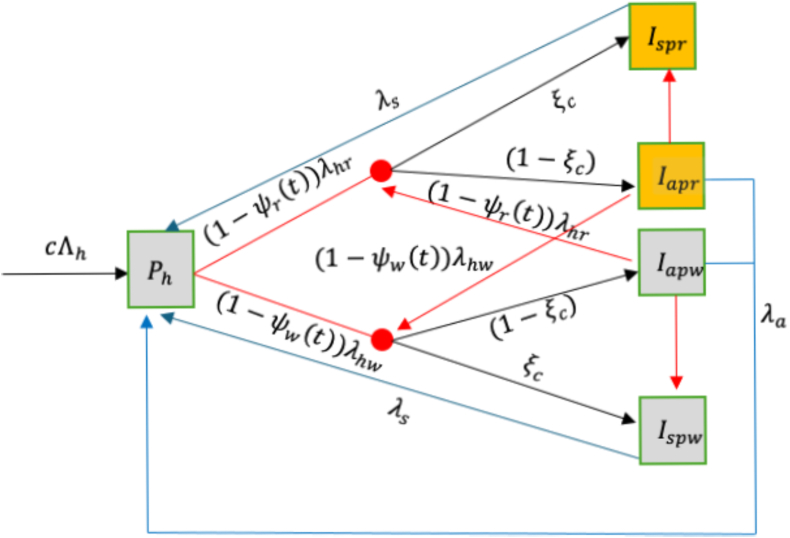
Schematic diagram for malaria infection within individuals receiving chemoprevention (children under 5).

The flow into and out of other compartments for this group (See Fig. 1) is the same as explained in the group without chemoprevention except for the probability by which these individuals develop symptoms and move into the symptomatic compartments which in this case is given as *ξ*_*c*_. Under this chemoprevention group, individuals age out after 5 years at the rate *ω* and join the mature group, which in this case is regarded to as the group of the individuals without chemoprevention. Natural death can occur from all compartments at a constant death rate of *μ*_*h*_. We do not consider the fitness cost in this model, so we assume that the force of infection for the resistant strain in both humans and mosquitoes is not reduced compared to that of the wild-type strain.

The mosquito population is recruited at the rate Λ_*v*_ and experiences natural mortality at a rate *μ*_*v*_. They are infected with either wild-type or resistant strain (not both) depending on whether the bite was on individual infected with wild-type or resistant strain. Upon being infected through the force of infections *λ*_*vw*_ corresponding to wild-type strain or *λ*_*vr*_ corresponding to the resistant strain, mosquitoes move to their strain specific incubation class. When the incubation period is over, mosquitoes progress to the infectious states depending on the strain they are infected with at the rate *ϵ.*

**Fig. 2:**
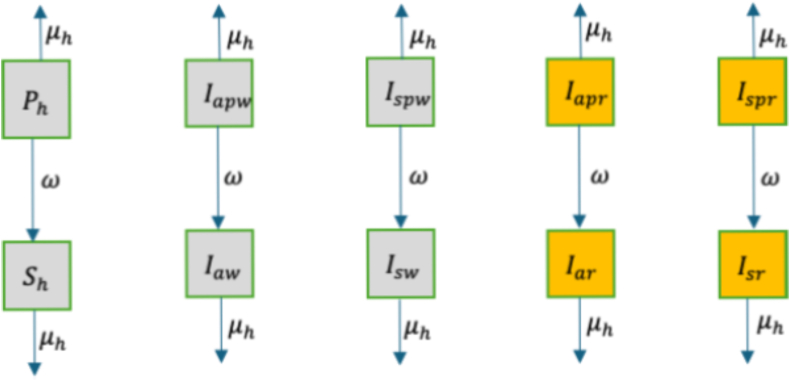
Transfer of individuals from the chemoprevention group to the non-chemoprevention group. In each state, individuals die naturally.

**Fig. 3:**
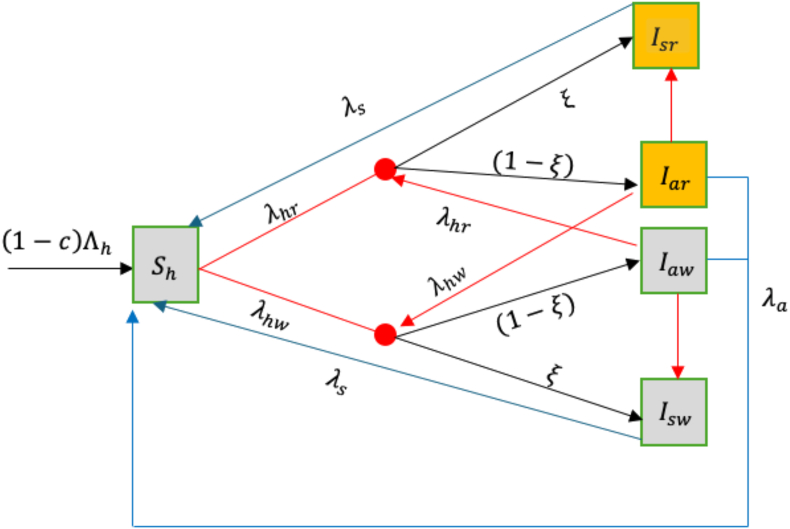
Schematic diagram for malaria infection for the individuals without chemoprevention.

Individuals with chemoprevention leave this group at the ageing rate *ω* to join individuals in elder group in Fig. 3 who are no longer eligible for receiving chemoprevention. We assume that these individuals do not retain some protection upon ageing because the drug protection does not last long enough until the individuals reach this stage. Transfer of individuals from this group is elaborated using Fig. 2.

For the human population dynamics, the model is governed by the differential equations;

(1) $$\begin{array}{ll} \frac{dS_{h}}{dt} & =\left(1-c\right)\Lambda_{h}N_{h}-\lambda_{hw}S_{h}-\lambda_{hr}S_{h}+\gamma_{s}\left(I_{sw}+I_{sr}\right)+\gamma_{a}\left(I_{aw}+I_{ar}\right)-\mu_{h}S_{h}+\omega P_{h},\\ \frac{dI_{sw}}{dt} & =\xi_{a}\lambda_{hw}\left(S_{h}+I_{ar}+I_{aw}\right)-\gamma_{s}I_{sw}-\mu_{h}I_{sw}+\omega I_{\mathrm{spw}},\\ \frac{dI_{aw}}{dt} & =\left(1-\xi_{a}\right)\lambda_{hw}\left(S_{h}+I_{ar}\right)-\lambda_{hr}I_{aw}-\xi_{a}\lambda_{hw}I_{aw}-\gamma_{a}I_{aw}-\mu_{h}I_{aw}+\omega I_{\mathrm{apw}},\\ \frac{dI_{sr}}{dt} & =\xi_{a}\lambda_{hr}\left(S_{h}+I_{ar}+I_{aw}\right)-\gamma_{s}I_{sr}-\mu_{h}I_{sr}+\omega I_{\mathrm{spr}},\\ \frac{dI_{ar}}{dt} & =\left(1-\xi_{a}\right)\lambda_{hr}\left(S_{h}+I_{aw}\right)-\lambda_{hw}I_{ar}-\xi_{a}\lambda_{hr}I_{ar}-\gamma_{a}I_{ar}-\mu_{h}I_{ar}+\omega I_{\mathrm{apr}},\\ \frac{dP_{h}}{dt} & =c\Lambda_{h}N_{h}-\left(1-\psi_{w}\right)\lambda_{hw}P_{h}-\left(1-\psi_{r}\right)\lambda_{hr}P_{h}+\gamma_{s}\left(I_{\mathrm{spw}}+I_{\mathrm{spr}}\right)+\gamma_{a}\left(I_{\mathrm{apw}}+I_{\mathrm{apr}}\right)\\ & \;-P_{h}\left(\mu_{h}+\omega \right),\\ \frac{dI_{\mathrm{spw}}}{dt} & =\xi_{c}\left(1-\psi_{w}\right)\lambda_{hw}\left(P_{h}+I_{\mathrm{apr}}+I_{\mathrm{apw}}\right)-\gamma_{s}I_{\mathrm{spw}}-I_{\mathrm{spw}}\left(\mu_{h}+\omega \right),\\ \frac{dI_{\mathrm{apw}}}{dt} & =\left(1-\xi_{c}\right)\lambda_{hw}\left(P_{h}+I_{\mathrm{apr}}\right)-\left(1-\psi_{r}\right)\lambda_{hr}I_{\mathrm{apw}}-\xi_{c}\left(1-\psi_{w}\right)\lambda_{hw}I_{\mathrm{apw}}-\gamma_{a}I_{\mathrm{apw}}\\ & \;-I_{\mathrm{apw}}\left(\mu_{h}+\omega \right),\\ \frac{dI_{\mathrm{spr}}}{dt} & =\xi_{c}\left(1-\psi_{r}\right)\lambda_{hr}\left(P_{h}+I_{\mathrm{apr}}+I_{\mathrm{apw}}\right)-\gamma_{s}I_{\mathrm{spr}}-I_{\mathrm{spr}}\left(\mu_{h}+\omega \right),\\ \frac{dI_{\mathrm{apr}}}{dt} & =\left(1-\xi_{c}\right)\lambda_{hr}\left(P_{h}+I_{\mathrm{apw}}\right)-\left(1-\psi_{w}\right)\lambda_{hw}I_{\mathrm{apr}}-\xi_{c}\left(1-\psi_{r}\right)\lambda_{hr}I_{\mathrm{apr}}-\gamma_{a}I_{\mathrm{apr}}\\ & \;-I_{\mathrm{apr}}\left(\mu_{h}+\omega \right). \end{array}$$

For the mosquito population dynamics, the model is governed by the differential equations;

(2) $$\begin{array}{ll} \frac{dS_{v}}{dt} & =\Lambda_{v}\left(t\right)N_{v}-\left(\lambda_{vw}+\lambda_{vr}\right)S_{v}-\mu_{v}S_{v},\\ \frac{dE_{vw}}{dt} & =\lambda_{vw}S_{v}-\epsilon E_{vw}-\mu_{v}E_{vw},\\ \frac{dE_{vr}}{dt} & =\lambda_{vr}S_{v}-\epsilon E_{vr}-\mu_{v}E_{vr},\\ \frac{dI_{vw}}{dt} & =\epsilon E_{vw}-\mu_{v}I_{vw},\\ \frac{dI_{vr}}{dt} & =\epsilon E_{vr}-\mu_{v}I_{vr}. \end{array}$$

Forces of infection are strain dependent. For humans, the forces of infection are expressed as

$$\lambda_{hw}=\frac{ab_{h}I_{vw}}{N_{h}},\text{ and }\lambda_{hr}=\frac{ab_{h}I_{vr}}{N_{h}},$$

for the wild-type and resistant strains, respectively. In mosquitoes, the forces of infection are expressed as

$$\lambda_{vw}=\frac{ab_{v}\left(I_{sw}+I_{aw}+I_{\mathrm{spw}}+I_{\mathrm{apw}}\right)}{N_{h}},\text{ for the wild}-\text{type strain and }\lambda_{vr}=\frac{ab_{v}\left(I_{sr}+I_{ar}+I_{\mathrm{spr}}+I_{\mathrm{apr}}\right)}{N_{h}},$$

for the resistant strain, Where; *a* is the mosquito biting rate, *b*_*h*_ is the probability that a bite results in transmission of sporozoites from an infected mosquito to a susceptible human and *b*_*v*_ is the probability that a bite results in transmission of gametocytes from an infected human to a susceptible mosquito.

## 3 Analysis of model

### 3.1 Invariance of the region, positivity and boundedness of the solution

For the epidemiological model to be meaningful, it is important to prove that all solutions with nonnegative initial data will remain nonnegative all the time. The model system for human population in system (1) and for the mosquito population in system (2) are epidemiologically and mathematically well posed in the domain

(3) $$\Omega =\Omega_{h}\times \Omega_{v}\subset R_{+}^{10}\times R_{+}^{5}$$

where $\Omega_{h}=\left\{\left(S_{h},I_{sw},I_{aw},I_{sr},I_{ar},P_{h},I_{\mathrm{spw}},I_{\mathrm{apw}},I_{\mathrm{spw}},I_{\mathrm{apr}}\right)\in R_{+}^{10}:N_{h}\leq \frac{\Lambda_{h}}{\mu_{h}}\right\}$ and $\Omega_{v}=\left\{\left(S_{v},E_{vw},E_{vr},I_{vw},I_{vr}\right)\in R_{+}^{5}:N_{v}\leq \frac{\Lambda_{v}}{\mu_{v}}\right\}$.

Note that Ω is invariant. The following theorem is used to determine the positivity and boundedness of the solution of systems in both humans and mosquitoes.

Theorem 1 *If* *S*_*h*_(0), *I*_*sw*_(0), *I*_*aw*_(0), *I*_*sr*_(0), *I*_*ar*_(0), *P*_*h*_(0), *I*_*spw*_(0), *I*_*apw*_(0), *I*_*spw*_(0), *I*_*apr*_(0) *are nonnegative, then so areS*_*h*_(*t*), *I*_*sw*_(*t*), *I*_*aw*_(*t*), *I*_*sr*_(*t*), *I*_*ar*_(*t*), *P*_*h*_(*t*), *I*_*spw*_(*t*), *I*_*apw*_(*t*), *I*_*spw*_(*t*), *I*_*apr*_(*t*) *for all time* *t* > 0*. Moreover,*

(4) $$\lim_{t\Rightarrow \infty }\text{sup}N_{h}\left(t\right)\leq \frac{\Lambda_{h}}{\mu_{h}}\text{ and}\lim_{t\Rightarrow \infty }\text{sup}N_{v}\left(t\right)\leq \frac{\Lambda_{v}}{\mu_{v}}.$$

*Furthermore, if* $N_{h}\left(0\right)\leq \frac{\Lambda_{h}}{\mu_{h}},\;then,N_{h}\left(t\right)\leq \frac{\Lambda_{h}}{\mu_{h}}$ *if* $N_{v}\left(0\right)\leq \frac{\Lambda_{v}}{\mu_{v}},\;then,N_{v}\left(t\right)\leq \frac{\Lambda_{v}}{\mu_{v}}$.

**Proof.** For the first part of the theorem it is enough to show that ${\frac{dx_{i}}{dt}}_{\left|x_{i}=0\right)}\geq 0\;$ where *x*_*i*_ is the state variable. If we consider the first equation of system (1) $\frac{dS_{h}}{dt}=\left(1-c\right)\Lambda_{h}N_{h}-\lambda_{hw}S_{h}-\lambda_{hr}S_{h}+\gamma_{s}\left(I_{sw}+I_{sr}\right)+\gamma_{s}\left(I_{aw}+I_{ar}\right)-\mu_{h}S_{h}+\omega P_{h}$ setting *S*_*h*_ = 0, we have, $\frac{dS_{h}}{dt}=\left(1-c\right)\Lambda_{h}N_{h}+\gamma_{s}\left(I_{sw}+I_{sr}\right)+\gamma_{a}\left(I_{aw}+I_{ar}\right)+\omega P_{h}\geq 0$. This can also be shown to be true for all equations in systems (1) and (2). To further analyze the malaria model, it is easier to work with a fractional of the population instead of the actual population, therefore, we scaled the population of each class in both the human population in system 1 and the mosquito population in system (2) by the total species population. Doing this will lead to the newly presented infections forces in humans such as *λ*_*hw*_ = *ab*_*h*_*mI*_*vw*_ for the wild-type strain and *λ*_*hr*_ = *ab*_*h*_*mI*_*vr*_ for the resistant strain, where $m=\frac{N_{v}}{N_{h}}$, which is defined as the number of mosquitoes per person.

For the second part of the proof, we add differential equations for human and mosquito population in the model system (1) and (2) to obtain

(5) $$\begin{array}{ll} \frac{dN_{h}}{dt} & =\Lambda_{h}-\mu_{h}N_{h},\\ \frac{dN_{v}}{dt} & =\Lambda_{v}-\mu_{v}N_{v}. \end{array}$$

From (5) when we consider the equation for the $\frac{dN_{h}}{dt}$ we have

(6) $$\Lambda_{h}-\mu_{h}N_{h}\leq \frac{dN_{h}}{dt}\leq \Lambda_{h}-\mu_{h}N_{h}.$$

Applying a standard comparison theorem in (6) as explained in Lakshmikantham et al. (1989), we obtain

(7) $$N_{h}\left(0\right)e^{-\mu_{h}t}+\frac{\Lambda_{h}}{\mu_{h}}\left(1-e^{-\mu_{h}t}\right)\leq N_{h}\left(t\right)\leq N_{h}\left(0\right)e^{-\mu_{h}t}+\frac{\Lambda_{h}}{\mu_{h}}\left(1-e^{-\mu_{h}t}\right).$$

This implies that

$$\frac{\Lambda_{h}}{\mu_{h}}\leq \lim_{t\Rightarrow \infty }\text{sup}N_{h}\left(t\right)\leq \lim_{t\Rightarrow \infty }\text{sup}N_{h}\left(t\right)\leq \frac{\Lambda_{h}}{\mu_{h}}.$$

Similarly, the same case can be made when we consider the equation for $\frac{dS_{v}}{dt}$ in (5). Moreover, if $N_{h}\left(0\right)\leq \frac{\Lambda_{h}}{\mu_{h}}$, then $N_{h}\left(t\right)\leq \frac{\Lambda_{h}}{\mu_{h}}$. This establishes the invariance of the domain Ω as required.

The model system presented by (1) and (2) is therefore epidemiologically feasible and mathematically well posed in the domain Ω.

### 3.2 Disease free equilibrium point (DFE)

Let

$$E_{0}^{*}=\left(S_{h}^{*},I_{sw}^{*},I_{aw}^{*},I_{sr}^{*},I_{ar}^{*},P_{h}^{*},I_{\mathrm{spw}}^{*},I_{\mathrm{apw}}^{*},I_{\mathrm{spr}}^{*},I_{\mathrm{apr}}^{*},S_{v}^{*},E_{vw}^{*},E_{vr}^{*},I_{vw}^{*},I_{vr}^{*}\right)$$

denote the equilibrium of the system described by the equations in systems 1 and 2. The system has a DFE

$$E_{0}^{*}=\left(S_{h}^{*},0,0,0,0,P_{h}^{*},0,0,0,0,S_{v}^{*},0,0,0,0\right),$$

where, $S_{h}^{*}=\frac{\Lambda_{h}\left(\mu_{h}+\omega -\mu_{h}c\right)}{\mu_{h}\left(\mu_{h}+\omega \right)}$, $P_{h}^{*}=\frac{c\Lambda_{h}}{\mu_{h}+\omega }$ and $S_{v}^{*}=\frac{\Lambda_{v}}{\mu_{v}}$.

### 3.3 The basic reproduction number

The basic reproduction number *R*_0_ is calculated from the twelve states compartments carrying the disease, *I*_*sw*_, *I*_*aw*_, *I*_*sr*_, *I*_*ar*_, *I*_*spw*_, *I*_*apw*_, *I*_*spr*_, *I*_*apr*_ for the humans and *E*_*vw*_, *E*_*vr*_, *I*_*vw*_, *I*_*vr*_ for the mosquitoes. Considering the infected compartments, the model system (1) and (2) can be written as;

(8) $$\begin{array}{ll} \frac{dI_{sw}}{dt} & =\xi_{a}ab_{h}mI_{vw}\left(S_{h}+I_{ar}+I_{aw}\right)-\gamma_{s}I_{sw}-\mu_{h}I_{sw}+\omega I_{\mathrm{spw}},\\ \frac{dI_{aw}}{dt} & =\left(1-\xi_{a}\right)ab_{h}mI_{vw}\left(S_{h}+I_{ar}\right)-ab_{h}mI_{vr}I_{aw}-\xi_{a}ab_{h}mI_{vw}I_{aw}-\gamma_{a}I_{aw}-\mu_{h}I_{aw}+\omega I_{\mathrm{apw}},\\ \frac{dI_{sr}}{dt} & =\xi_{a}ab_{h}mI_{vr}\left(S_{h}+I_{ar}+I_{aw}\right)-\gamma_{s}I_{sr}-\mu_{h}I_{sr}+\omega I_{\mathrm{spr}},\\ \frac{dI_{ar}}{dt} & =\left(1-\xi_{a}\right)ab_{h}mI_{vr}\left(S_{h}+I_{aw}\right)-ab_{h}mI_{vw}I_{ar}-\xi_{a}ab_{h}mI_{vr}I_{ar}-\gamma_{a}I_{ar}-\mu_{h}I_{ar}+\omega I_{\mathrm{apr}},\\ \frac{dI_{\mathrm{spw}}}{dt} & =\xi_{c}\left(1-\psi_{w}\right)ab_{h}mI_{vw}\left(P_{h}+I_{\mathrm{apr}}+I_{\mathrm{apw}}\right)-\gamma_{s}I_{\mathrm{spw}}-I_{\mathrm{spw}}\left(\mu_{h}+\omega \right),\\ \frac{dI_{\mathrm{apw}}}{dt} & =\left(1-\xi_{c}\right)ab_{h}mI_{vw}\left(P_{h}+I_{\mathrm{apr}}\right)-\left(1-\psi_{r}\right)ab_{h}mI_{vr}I_{\mathrm{apw}}-\xi_{c}\left(1-\psi_{w}\right)ab_{h}mI_{vw}I_{\mathrm{apw}}-\gamma_{a}I_{\mathrm{apw}}\\ & \;-I_{\mathrm{apw}}\left(\mu_{h}+\omega \right),\\ \frac{dI_{\mathrm{spr}}}{dt} & =\xi_{c}\left(1-\psi_{r}\right)ab_{h}mI_{vr}\left(P_{h}+I_{\mathrm{apr}}+I_{\mathrm{apw}}\right)-\gamma_{s}I_{\mathrm{spr}}-I_{\mathrm{spr}}\left(\mu_{h}+\omega \right),\\ \frac{dI_{\mathrm{apr}}}{dt} & =\left(1-\xi_{c}\right)ab_{h}mI_{vr}\left(P_{h}+I_{\mathrm{apw}}\right)-\left(1-\psi_{w}\right)ab_{h}mI_{vw}I_{\mathrm{apr}}-\xi_{c}\left(1-\psi_{r}\right)ab_{h}mI_{vr}I_{\mathrm{apr}}-\gamma_{a}I_{\mathrm{apr}}\\ & \;-I_{\mathrm{apr}}\left(\mu_{h}+\omega \right),\\ \frac{dE_{vw}}{dt} & =ab_{v}\left(I_{sw}+I_{aw}+I_{\mathrm{spw}}+I_{\mathrm{apw}}\right)S_{v}-\epsilon E_{vw}-\mu_{v}E_{vw},\\ \frac{dE_{vr}}{dt} & =ab_{v}\left(I_{sr}+I_{ar}+I_{\mathrm{spr}}+I_{\mathrm{apr}}\right)S_{v}-\epsilon E_{vr}-\mu_{v}E_{vr}\\ \frac{dI_{vw}}{dt} & =\epsilon E_{vw}-\mu_{v}I_{vw},\\ \frac{dI_{vr}}{dt} & =\epsilon E_{vr}-\mu_{v}I_{vr}. \end{array}$$

From the model system (8), *R*_0_ can be determined considering compartments with resistant and wild-type strains separately. The total *R*_0_ would therefore be given as the sum of $R_{0}^{r}$ and $R_{0}^{w}$; thus $R_{0}=R_{0}^{r}+R_{0}^{w}$. To compute *R*_0_ we use the next generation matrix approach as explained in Van den Driessche and Watmough (2002). For $R_{0}^{r}$, we consider the system

(9) $$\begin{array}{ll} \frac{dI_{sr}}{dt} & =\xi_{a}ab_{h}mI_{vr}\left(S_{h}+I_{ar}+I_{aw}\right)-\gamma_{s}I_{sr}-\mu_{h}I_{sr}+\omega I_{\mathrm{spr}},\\ \frac{dI_{ar}}{dt} & =\left(1-\xi_{a}\right)ab_{h}mI_{vr}\left(S_{h}+I_{aw}\right)-ab_{h}mI_{vw}I_{ar}-\xi_{a}ab_{h}mI_{vr}I_{ar}-\gamma_{a}I_{ar}-\mu_{h}I_{ar}+\omega I_{\mathrm{apr}},\\ \frac{dI_{\mathrm{spr}}}{dt} & =\xi_{c}\left(1-\psi_{r}\right)ab_{h}mI_{vr}\left(P_{h}+I_{\mathrm{apr}}+I_{\mathrm{apw}}\right)-\gamma_{s}I_{\mathrm{spr}}-I_{\mathrm{spr}}\left(\mu_{h}+\omega \right),\\ \frac{dI_{\mathrm{apr}}}{dt} & =\left(1-\xi_{c}\right)ab_{h}mI_{vr}\left(P_{h}+I_{\mathrm{apw}}\right)-\left(1-\psi_{w}\right)ab_{h}mI_{vw}I_{\mathrm{apr}}-\xi_{c}\left(1-\psi_{r}\right)ab_{h}mI_{vr}I_{\mathrm{apr}}-\gamma_{a}I_{\mathrm{apr}}\\ & \;-I_{\mathrm{apr}}\left(\mu_{h}+\omega \right),\\ \frac{dE_{vr}}{dt} & =ab_{v}\left(I_{sr}+I_{ar}+I_{\mathrm{spr}}+I_{\mathrm{apr}}\right)S_{v}-\epsilon E_{vr}-\mu_{v}E_{vr},\\ \frac{dI_{vr}}{dt} & =\epsilon E_{vr}-\mu_{v}I_{vr}. \end{array}$$

We construct the matrices $F_{i}$ and $V_{i}$ for system (7) then, by differentiating F and V partially with respect to *I*_*sr*_, *I*_*ar*_, *I*_*spr*_, *I*_*apr*_, *E*_*vr*_ and *I*_*vr*_ at DFE point we get,

(10) $$F=\left(\begin{array}{llllll} 0 & 0 & 0 & 0 & 0 & \frac{am\xi_{a}b_{h}\Lambda_{h}\left(-c\mu_{h}+\mu_{h}+\omega \right)}{\mu_{h}\left(\mu_{h}+\omega \right)}\\ 0 & 0 & 0 & 0 & 0 & \frac{am\left(1-\xi_{a}\right)b_{h}\Lambda_{h}\left(-c\mu_{h}+\mu_{h}+\omega \right)}{\mu_{h}\left(\mu_{h}+\omega \right)}\\ 0 & 0 & 0 & 0 & 0 & \frac{acmb_{h}\xi_{c}\Lambda_{h}\left(1-\psi_{r}\right)}{\mu_{h}+\omega }\\ 0 & 0 & 0 & 0 & 0 & \frac{acmb_{h}\left(1-\xi_{c}\right)\Lambda_{h}}{\mu_{h}+\omega }\\ \frac{ab_{v}\Lambda_{v}}{\mu_{v}} & \frac{ab_{v}\Lambda_{v}}{\mu_{v}} & \frac{ab_{v}\Lambda_{v}}{\mu_{v}} & \frac{ab_{v}\Lambda_{v}}{\mu_{v}} & 0 & 0\\ 0 & 0 & 0 & 0 & 0 & 0 \end{array}\right)$$

and

(11) $$V=\left(\begin{array}{llllll} \mu_{h}+\gamma_{s} & 0 & -\omega & 0 & 0 & 0\\ 0 & \gamma_{a}+\mu_{h} & 0 & \omega & 0 & 0\\ 0 & 0 & \mu_{h}+\gamma_{s}+\omega & 0 & 0 & 0\\ 0 & 0 & 0 & \gamma_{a}+\mu_{h}+\omega & 0 & 0\\ 0 & 0 & 0 & 0 & \epsilon +\mu_{v} & 0\\ 0 & 0 & 0 & 0 & -\epsilon & \mu_{v} \end{array}\right)$$

The basic reproduction number *R*_0_ of the model system (7) is given as the greatest eigenvalues (spectral radius) of the next generation matrix of *FV*^−1^. The basic reproductive number for the resistant strain $\left(R_{0}^{r}\right)$ is therefore given as

$$R_{0}^{r}=\sqrt{\frac{A_{1}\left(A_{2}-A_{5}\right)}{A_{4}}}$$

where,

$$\begin{array}{ll} A_{1} & =a^{2}m\epsilon \;b_{h}b_{v}\Lambda_{h}\Lambda_{v},\\ A_{2} & =\mu_{h}^{3}+\left(2\omega +\gamma_{a}+\gamma_{s}+\gamma_{a}\xi_{a}+c\gamma_{s}\xi_{a}+2c\omega \xi_{c}+c\gamma_{a}\xi_{c}\right)\;\mu_{h}^{2}\\ & \;+\left(\omega^{2}+\omega \gamma_{a}+2\omega \gamma_{s}+\gamma_{a}\gamma_{s}+\gamma_{a}^{2}\xi_{a}+2\omega \gamma_{a}\xi_{a}+c\omega \gamma_{s}\xi_{a}+c\gamma_{a}\gamma_{s}\xi_{a}\right)\\ & \left(\;+c\gamma_{a}^{2}\xi_{c}+c\omega \gamma_{a}\xi_{c}+c\omega \gamma_{s}\xi_{c}\right)\;\mu_{h}\\ & \;+\omega^{2}\gamma_{s}+\omega \gamma_{a}\gamma_{s}+\omega \gamma_{a}^{2}\xi_{a}+\omega^{2}\gamma_{a}\xi_{a},\\ A_{4} & =\mu_{v}^{2}\;\mu_{h}\;\left(\omega +\mu_{h}\right)\left(\gamma_{a}+\mu_{h}\right)\left(\omega +\gamma_{a}+\mu_{h}\right)\left(\gamma_{s}+\mu_{h}\right)\left(\epsilon +\mu_{v}\right),\\ A_{5} & =c\xi_{c}\psi_{r}\;\mu_{h}^{3}+\left(2c\omega +c\gamma_{a}\xi_{a}+\gamma_{s}\xi_{a}+c\gamma_{s}\xi_{c}+c\omega \xi_{c}\psi_{r}+2c\gamma_{a}\xi_{c}\psi_{r}\right)\;\mu_{h}^{2}\\ & \;+\left(2c\omega \gamma_{s}+c\gamma_{a}^{2}\xi_{a}+c\omega \gamma_{a}\xi_{a}+2\omega \gamma_{s}\xi_{a}+\gamma_{a}\gamma_{s}\xi_{a}+c\gamma_{a}\gamma_{s}\xi_{c}+c\gamma_{a}^{2}\xi_{c}\psi_{r}\right)\\ & \left(\;+c\omega \gamma_{a}\xi_{c}\psi_{r}\right)\;\mu_{h}\\ & \;+\omega^{2}\gamma_{s}\xi_{a}+\omega \gamma_{a}\gamma_{s}\xi_{a}. \end{array}$$

For the $R_{0}^{w}$, we consider the system

(12) $$\begin{array}{ll} \frac{dI_{sw}}{dt} & =\xi_{a}ab_{h}mI_{vw}\left(S_{h}+I_{ar}+I_{aw}\right)-\gamma_{s}I_{sw}-\mu_{h}I_{sw}+\omega I_{\mathrm{spw}},\\ \frac{dI_{aw}}{dt} & =\left(1-\xi_{a}\right)ab_{h}mI_{vw}\left(S_{h}+I_{ar}\right)-ab_{h}mI_{vr}I_{aw}-\xi_{a}ab_{h}mI_{vw}I_{aw}-\gamma_{a}I_{aw}-\mu_{h}I_{aw}+\omega I_{\mathrm{apw}},\\ \frac{dI_{\mathrm{spw}}}{dt} & =\xi_{c}\left(1-\psi_{w}\right)ab_{h}mI_{vw}\left(P_{h}+I_{\mathrm{apr}}+I_{\mathrm{apw}}\right)-\gamma_{s}I_{\mathrm{spw}}-I_{\mathrm{spw}}\left(\mu_{h}+\omega \right),\\ \frac{dI_{\mathrm{apw}}}{dt} & =\left(1-\xi_{c}\right)ab_{h}mI_{vw}\left(P_{h}+I_{\mathrm{apr}}\right)-\left(1-\psi_{r}\right)ab_{h}mI_{vr}I_{\mathrm{apw}}-\xi_{c}\left(1-\psi_{w}\right)ab_{h}mI_{vw}I_{\mathrm{apw}}-\gamma_{a}I_{\mathrm{apw}}\\ & \;-I_{\mathrm{apw}}\left(\mu_{h}+\omega \right),\\ \frac{dE_{vw}}{dt} & =ab_{v}\left(I_{sw}+I_{aw}+I_{\mathrm{spw}}+I_{\mathrm{apw}}\right)S_{v}-\epsilon E_{vw}-\mu_{v}E_{vw},\\ \frac{dI_{vw}}{dt} & =\epsilon E_{vw}-\mu_{v}I_{vw}. \end{array}$$

We construct the matrices $F_{i}$ and $V_{i}$ for system (12) then, by differentiating F and V partially with respect to *I*_*sw*_, *I*_*aw*_, *I*_*spw*_, *I*_*apw*_, *E*_*vw*_ and *I*_*vw*_ at DFE point we get,

(13) $$F=\left(\begin{array}{llllll} 0 & 0 & 0 & 0 & 0 & \frac{am\xi_{a}b_{h}\Lambda_{h}\left(-c\mu_{h}+\mu_{h}+\omega \right)}{\mu_{h}\left(\mu_{h}+\omega \right)}\\ 0 & 0 & 0 & 0 & 0 & \frac{am\left(1-\xi_{a}\right)b_{h}\Lambda_{h}\left(-c\mu_{h}+\mu_{h}+\omega \right)}{\mu_{h}\left(\mu_{h}+\omega \right)}\\ 0 & 0 & 0 & 0 & 0 & \frac{acmb_{h}\xi_{c}\Lambda_{h}\left(1-\psi_{w}\right)}{\mu_{h}+\omega }\\ 0 & 0 & 0 & 0 & 0 & \frac{acmb_{h}\left(1-\xi_{c}\right)\Lambda_{h}}{\mu_{h}+\omega }\\ \frac{ab_{v}\Lambda_{v}}{\mu_{v}} & \frac{ab_{v}\Lambda_{v}}{\mu_{v}} & \frac{ab_{v}\Lambda_{v}}{\mu_{v}} & \frac{ab_{v}\Lambda_{v}}{\mu_{v}} & 0 & 0\\ 0 & 0 & 0 & 0 & 0 & 0 \end{array}\right)$$

and

(14) $$V=\left(\begin{array}{llllll} \mu_{h}+\gamma_{s} & 0 & -\omega & 0 & 0 & 0\\ 0 & \gamma_{a}+\mu_{h} & 0 & \omega & 0 & 0\\ 0 & 0 & \mu_{h}+\gamma_{s}+\omega & 0 & 0 & 0\\ 0 & 0 & 0 & \gamma_{a}+\mu_{h}+\omega & 0 & 0\\ 0 & 0 & 0 & 0 & \epsilon +\mu_{v} & 0\\ 0 & 0 & 0 & 0 & -\epsilon & \mu_{v} \end{array}\right)$$

The basic reproduction number *R*_0_ of the model system (12) is given as the greatest eigenvalue (spectral radius) of the next generation matrix of *FV*^−1^. The basic reproductive number for the wild-type strain $\left(R_{0}^{w}\right)$ is therefore given as

$$R_{0}^{w}=\sqrt{\frac{A_{1}\left(A_{2}-A_{3}\right)}{A_{4}}}$$

where;

$$\begin{array}{ll} A_{3} & =c\xi_{c}\psi_{w}\;\mu_{h}^{3}+\left(2c\omega +c\gamma_{a}\xi_{a}+\gamma_{s}\xi_{a}+c\gamma_{s}\xi_{c}+c\omega \xi_{c}\psi_{w}+2c\gamma_{a}\xi_{c}\psi_{w}\right)\;\mu_{h}^{2}\\ & \;+\left(2c\omega \gamma_{s}+c\gamma_{a}^{2}\xi_{a}+c\omega \gamma_{a}\xi_{a}+2\omega \gamma_{s}\xi_{a}+\gamma_{a}\gamma_{s}\xi_{a}+c\gamma_{a}\gamma_{s}\xi_{c}+c\gamma_{a}^{2}\xi_{c}\psi_{w}\right)\\ & \left(\;+c\omega \gamma_{a}\xi_{c}\psi_{w}\right)\;\mu_{h}\\ & \;+\omega^{2}\gamma_{s}\xi_{a}+\omega \gamma_{a}\gamma_{s}\xi_{a}. \end{array}$$

The basic reproduction number for the model system (8), comprises two components, which are the basic reproduction numbers for the resistant strain $R_{0}^{r}$ and for the wild-type strain $R_{0}^{w}$, thus $R_{0}=R_{0}^{r}+R_{0}^{w}$. The expressions *A*_5_ and *A*_3_ show the difference between the basic reproduction number for the resistant and wild-type strains, respectively. When chemoprevention coverage is 0%, $R_{0}^{r}=\;R_{0}^{w}$, making the basic reproduction number *R*_0_ for the model system (8) representing the class for the individuals without chemoprevention. Further analysis to understand how some of the selected parameters influence the spread of resistance is presented in the following section.

## 4 Numerical results

### 4.1 Simulations and model parameters

To understand how chemoprevention coverage and the number of treatment cycles impact the spread of *dhps* A581G mutation, an age-structured deterministic model was calibrated and simulated in R version 4.2.2. This model considers the setting where *dhps* K540E mutation is most prevalent and already near fixation (it's prevalence is more than 75%, this situation mostly represents East African settings) and thus regarded as wild-type in this model, and the prevalence of A581G mutation is emerging. Coverage levels of chemoprevention using SP were varied between 0and 80% in the interval of 15%. In the first scenario, three cycles of chemoprevention were administered every thirty (30) days during March, April, and May, and in the second scenario, five cycles of chemoprevention were administered every thirty (30) days in February, March, April, May and June.

We computed human birth rate per day using the formula

$$\Lambda_{h}=\frac{\text{Number of live births per }1000\text{ people per year}}{1000\text{ people}}\times \frac{1\text{ year}}{356\text{ days}}$$

We used $\frac{35.8\text{ births}}{1000\text{ people}}$ to compute the human birth rate. In this model, we assume that human birth occurs at the rate Λ_*h*_ and natural death occurs in each class at the rate *μ*_*h*_ and the two rates are equal. This assumption leads to a constant population in our model.

The recruitment rate for mosquitoes Λ_*v*_ depends on seasonality. This is computed using a cosine function that is given as

$$\Lambda_{v}\left(t\right)=\left(A\cos\left(\frac{2\pi t}{365}+\phi \right)+1\right)\mu_{v}$$

We scale the seasonal factors relative to the mosquito death rate. The amplitude value A is set to 0.2 and in this case is used to control how much the mosquito birth rate varies over the year and in this case it is taken to be ±0.2. When we consider March to May as the high rain months coinciding with the highest malaria transmission season, we take into account a one-month lag for the mosquito population to peak. This function is used to make sure that, throughout we maintain a stable mosquito population by preventing explosion or collapse.

More of the model parameters used in the simulation are presented in Table 1.

**Table 1:** Parameter name (symbols), their description, values, and source(s).

Parameter (symbol)	Value	Source(s)
Mean duration of protection against the wild-type strain (*η*_*w*_)	16.5(11.2 − 37.4) days	Mousa et al. (2025)
Mean duration of protection against resistant strain (*η*_*r*_)	11.7(8.0 − 21.9) days	Mousa et al. (2025)
Weibull shape parameter of protection against the wild-type strain (*w*_*w*_)	5	Mousa et al. (2024)
Weibull shape parameter of protection against resistant strain (*w*_*r*_)	5	Mousa et al. (2024)
Weibull scale parameter of protection against the wild-type strain *λ*_*w*_	$\frac{\eta_{w}}{\Gamma \left(1+\frac{1}{w_{w}}\right)}$	Calculated
Weibull scale parameter of protection against resistant strain *λ*_*r*_	$\frac{\eta_{r}}{\Gamma \left(1+\frac{1}{w_{r}}\right)}$	Calculated
Probability of drug protection against the wild-type strain (*ψ*_*w*_)	$e^{-{\left(\frac{t}{\lambda_{w}}\right)}^{w_{w}}}$	Calculated
Probability of drug protection against resistant parasite (*ψ*_*r*_)	$e^{-{\left(\frac{t}{\lambda_{r}}\right)}^{w_{r}}}$	Calculated
Chemoprevention coverage (*c*)	(0 − 80%)	-
Proportion of children who develop symptoms (*ξ*_*c*_)	0.5	Prudhomme O'Meara et al. (2006)
Proportion of adults who develop symptoms (*ξ*_*a*_)	0.25	Prudhomme O'Meara et al. (2006)
Recovery rate for untreated individuals (*λ*_*a*_)	1/120day^−1^	(Cairns et al., 2011)
Recovery rate from symptomatic and treated individuals (*λ*_*s*_)	1/5day^−1^	Prudhomme O'Meara et al. (2006)
Mosquito biting rate (*a*)	0.33(0.01 − 1.0)day^−1^	(Chitnis et al., 2008)
Probability of transmission of infection from infectious mosquito to a susceptible human (*b*_*h*_)	0.2(0.010 − 0.27)	(Chitnis et al., 2008)
Probability of transmission of infection from infectious human to a susceptible mosquito (*b*_*v*_)	0.4(0.072 − 0.64)	(Chitnis et al., 2008)
Mosquito death rate (*μ*_*v*_)	0.0714(0.0010 − 0.10)day^−1^	(Chitnis et al., 2008)

### 4.2 Results

**Fig. 4:**
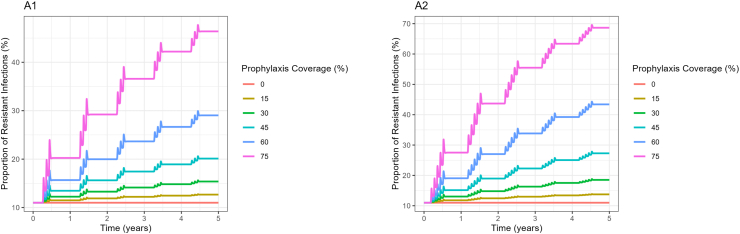
Proportion of resistant infections over time based on the number of treatment cycles. A1 shows the proportion of resistant infections over time when three cycles of chemoprevention are given and A2 shows the proportion of resistant infections over time when there are five cycles of chemoprevention.

Fig. 4 shows the proportion of resistant infections over time. In particular, A1 shows the proportion of resistant infections when three cycles of chemoprevention are administered at different coverage levels, while A2 shows the proportion of resistant infections when five cycles of chemoprevention are administered at different coverage levels. When chemoprevention coverage is very low in both scenarios, the proportion of resistant infections is also very low. Both graphs in Fig. 4 show that the proportion of resistant infections at chemoprevention coverage 0% remains constant throughout the simulation period. This explains how coverage of chemoprevention doses can accelerate the selection of the resistant strains leading to its spread. At the coverage of 0% there is no drug pressure, thus the prevalence of resistant infections in the population remains constant throughout. At 45% coverage, for example; the proportion of resistant infections in both A1 and A2 increases from 11% to 20.6% in A1 and 27.8% in A2, see Table 2. These results suggest that both chemoprevention coverage levels and chemoprevention treatment cycles per year influence the spread of *dhps* A581G mutation. A study by Masserey, Lee, et al. (2024) that estimated the impact of SMC on the spread of *dhfr* and *dhps* quintuple mutant resistant to sulphadoxine-pyrimethamine also confirms that chemoprevention coverage levels, among others, promote the spread of mutants. This study looked at the different mutation but still serves as an important way to confirm how chemoprevention coverage levels influence the spread of resistant mutations in the population. Other modeling studies also suggest that intermittent preventive treatments (IPT), and IPT dose frequency promote the spread of drug resistance (Manore et al., 2019; Teboh-Ewungkem et al., 2015). A non modeling study also confirms that chemoprevention in Mali was associated with the increase of *pfdhfr-pfdhps* quintuple mutant genotype among the children receiving chemoprevention compared to those who did not receive chempoprevention (Maiga et al., 2016).

**Table 2:** Proportion of resistant infections under different coverage levels for the 3 treatment cycles at the end of each year (%).

Coverage in %	Baseline	Y1	Y2	Y3	Y4	Y5
0	11.0	11.0	11.0	11.0	11.0	11.0
15	11.0	11.5	11.9	12.2	12.5	12.7
30	11.0	12.2	13.3	14.1	14.8	15.4
45	11.0	13.5	15.6	17.4	18.9	20.1
60	11.0	15.7	20.0	23.7	26.7	29.0
75	11.0	20.2	29.2	36.6	42.2	46.4

To better understand the influence of the number of cycles and coverage levels on the spread of *dhps* A581G mutation, we first performed a two way-anova to assess the effects of the number of treatment cycles and coverage levels on the spread of resistance using the following formula

$$y_{ij}=\mu +\tau_{i}+\beta_{j}+{\left(\tau \beta \right)}_{ij}+\epsilon_{\mathrm{ijk}},$$

where, *μ* is the baseline resistance level, *τ*_*i*_ is the effect due to the number of treatment cycles, *β*_*j*_ is the effect due to coverage levels, (*τβ*)_*ij*_ is the effect due to any interaction between the number of treatment cycles and the coverage levels. We also performed a partial $\eta_{P}^{2}$ given as $\frac{SS_{\mathrm{effect}}}{SS_{\mathrm{effect}}+SS_{\mathrm{error}}}$ to quantify how the number of treatment cycles and the coverage levels influence the spread of the resistant mutation while controlling for other variables in the model, where *SS* stands for sum of squares.

The results in Table 4 show that both the number of treatment cycles and coverage levels significantly influence the spread of resistance. There is a significant effect of the number of treatment cycles on the spread of resistance, $F\left(1,21908\right)=2237.94,p<.001,\eta_{P}^{2}=0.093$. This indicates that 9.3% of the spread of resistance 0.093(95% CI: 0.09 − 1.00) is influenced by the number of treatment cycles. Chemoprevention coverages show the strongest effect on the spread of resistance, $F\left(1,21908\right)=30231.49,p<.001,\eta_{P}^{2}=0.580$. This implies that 58% of the spread of resistance 0.580(95% CI: 0.57 − 1.00) is influenced by the coverage levels, making coverage levels the most influential factor affecting the spread of resistance. The interaction between the number of treatment cycles and chemoprevention coverages. This indicates that the effect of treatment cycles on the spread of resistance depends on the chemoprevention coverages $F\left(1,21908\right)=2170.34,p<.001,\eta_{P}^{2}=0.090$. This shows that, 9.0% of the spread of resistance 0.090(95% CI: 0.08 − 1.00) is influenced by the interaction between the number of treatment cycles and the chemoprevention coverage.

**Table 3:** Proportion of resistant infections under different coverage levels for the 5 treatment cycles at the end of each year (%).

Coverage in %	Baseline	Y1	Y2	Y3	Y4	Y5
0	11.0	11.0	11.0	11.0	11.0	11.0
15	11.0	12.0	12.4	13.0	13.4	13.8
30	11.0	13.0	14.8	16.3	17.5	18.5
45	11.0	15.1	19.0	22.3	25.0	27.3
60	11.0	19.0	27.0	33.8	39.2	43.0
75	11.0	27.5	43.7	55.5	63.4	68.7

**Table 4:** Effect sizes.

Effect	*df*_1_	*df*_2_	F	p	$\eta_{P}^{2}$
Cycles	1	21908	2237.95	0.00	0.093
Coverage	1	21908	30231.49	0.00	0.580
Cycles:Coverage	1	21908	2170.34	0.00	0.090

**Fig. 5:**
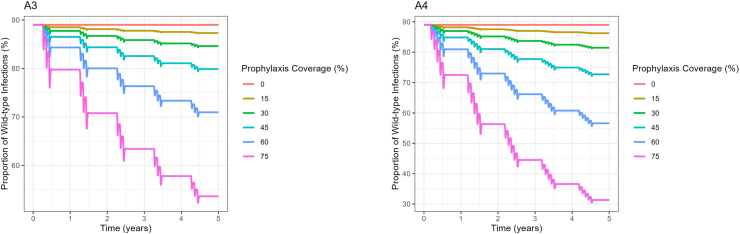
Proportion of the K540E infections over time based on the number of treatment cycles. A3 shows the proportion of the K540E infections over time when three cycles of chemoprevention are given and A4 shows the proportion of the K540E infections over time when there are five cycles of chemoprevention.

The results in Fig. 5 show the proportion of wild-type infections over time. A3 shows the proportion of the K540E infections when three cycles of chemoprevention are administered at different coverage levels, and A2 shows the proportion of the K540E infections when five cycles of chemoprevention are administered at different coverage levels. Higher levels of chemoprevention coverage reduce the proportion of wild-type infections over time. These results suggest that higher levels of chemoprevention coverage are more effective in reducing the proportion of wild-type infections in the population over time.

The number of clinical malaria cases averted increases with the increase in chemoprevention coverage. More clinical cases are averted with high levels of chemoprevention coverage compared to lower levels of chemoprevention coverage. However, the number of clinical cases averted varies slightly depending on the number of chemoprevention treatment cycles per year. Despite the presence of *dhps* A 581G mutation, chemoprevention using SP could still slightly help to avert malaria in children younger than 5 years. A modeling study by Manore et al. (2019) also confirms that Intermittent preventive treatment (IPT) plays an important role in averting malaria deaths when administered in holoendemic malaria regions.

## 5 Discussion

This study develops a deterministic age-structured compartmental model to investigate how chemoprevention coverage levels and the number of treatment cycles influence the spread of *dhps* A581G mutation. The model through simulations, evaluates two main scenarios of varying the levels of coverage of chemoprevention from 0% to 80% in the interval of 15% and increasing the number of treatment cycles for chemoprevention from three to five treatment cycles per year in settings where *dhps* K540E mutation is most prevalent and already near fixation, thus regarded as the wild-type strain in this model and the prevalence of *dhps* A581G mutation is emerging. Our model was able to predict that the spread of *dhps* A581G mutation could increase if both the levels of coverage of chemoprevention and the number of treatment cycles per year are increased.

The study shows that the increase in chemoprevention coverage levels influences the spread of resistant infections over time. With low chemoprevention coverage levels, the proportion of resistant infections remains low, while with high chemoprevention coverage levels, the proportion of resistant infections increases over time. It is also seen that, both the numbers of treatment cycles and chemoprevention coverage levels influence the spread of resistance. When the number of treatment cycles is increased from three to five cycles, the increase in the prevalence of resistant infections is also observed. From Table 2, Table 3, it is observed that, at 0% coverage of chemoprevention, the prevalence of resistant infections remain constant at 11% throughout. When both chemoprevention coverage level and treatment cycles are increased, the prevalence of resistant infections increases. These results demonstrate that high coverage and the number of treatment cycles can accelerate the selection of resistant strains.

Further analysis was performed to quantify the extent to which both the chemoprevention coverage levels, and the number of treatment cycles influence the spread of resistant infections over time. This was done by performing a partial eta squared while treating other variables in the model as constants. The results in Table 4 further suggest that both chemoprevention coverage and the number of treatment cycles significantly influence the spread of resistance. It was shown that 58.0% of the spread of resistance was explained by the chemoprevention coverage level, making coverage level the most influential factor affecting the spread of resistance. The number of treatment cycles per year also matters, but its influence on the spread of resistance was relatively smaller, only 9.3% of the spread of resistance was explained by the number of treatment cycles. Other studies also confirm that high coverage levels and frequent chemopreventive doses can accelerate the selection of resistant strain (Maiga et al., 2016; Manore et al., 2019; Masserey, Lee, et al., 2024; Teboh-Ewungkem et al., 2015).

**Fig. 6:**
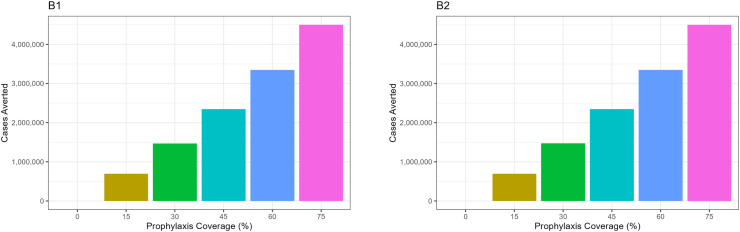
Number of clinical cases averted based on the number of treatment cycles. B1 shows the number of cases averted with three cycles of chemoprevention, and B2 shows the number of cases averted when there are five cycles of chemoprevention.

The number of clinical malaria cases averted, increased with the increase of chemoprevention coverage levels. More clinical malaria cases were averted with high chemoprevention coverage compared to when coverage was low. In Fig. 6 it is seen that, with high chemoprevention coverage levels, a large number of clinical malaria cases were averted.

Our model has some limitations, which should be acknowledged. We did not take into account other interventions in place, such as the use of LLINs, which have played a major role in reducing malaria cases. We also did not take into account the co-infection of strains in this model. To get more insight on the spread of SP-resistant mutations, and to better quantify the number of cases averted and other benefits of SP despite the presence of mutations, future studies should consider relaxing some of the assumptions considered in this model. Other interventions, such as vector-based interventions, should be taken into consideration; this could affect the model results and, in turn, the rate at which resistant mutations spread. We also did not consider the fitness cost of resistance, which might also affect the spread of resistant mutations. Relaxing these assumptions might slightly affect these results, especially with the spread of resistant mutations and the number of cases averted after administering chemoprevention using SP.

The analysis from this model focused on the influence of chemoprevention coverage levels and the number of treatment cycles on the spread of *dhps* A581G mutation. This was done using a partial eta squared measure, which excluded variance accounted for by other factors. Our model provides a structured and theory-driven approach to examine the relative influence of the key variables within a complex system, and such models are important in quantifying the effect sizes of different variables under controlled assumptions. We were able to demonstrate how both chemoprevention coverage levels and the number of chemoprevention treatment cycles using SP influence the spread of the *dhps* A581G mutation. We used partial eta squared to determine the size effects of these variables while controlling for other variables. This model was calibrated using data from various literature sources. Given the current real data limitations, our aim remains exploratory and a theory-driven deterministic model rather than predictive; however, incorporating real data for calibration and validation in future studies would further strengthen the applicability and generalizability of this model.

## 6 Conclusion

In this study, we developed a deterministic age-structured compartmental model to investigate how chemoprevention coverage levels and the number of treatment cycles influence the spread of *dhps* A581G mutation. The results of this model suggest that both chemoprevention coverage levels and the number of treatment cycles influence in a different way the spread of *dhps* A581G mutation, with coverage levels being the most influential factor for the spread of resistant parasites. Despite the presence of *dhps* A581G mutation, model results suggest that chemoprevention using SP still, in a way, remains effective in averting clinical malaria cases, especially when the chemoprevention coverage levels are higher compared to when chemoprevention coverage levels are low.

Chemoprevention using SP influences the spread of the *dhps* A581G mutation. Careful policy planning, decisions, and ongoing molecular surveillance are essential to optimize the benefits of chemoprevention using SP while minimizing the risk of the spread of the *dhps* A581G mutation.

## CRediT authorship contribution statement

**Nicholaus Mziray:** Writing – review & editing, Writing – original draft, Methodology, Conceptualization. **Lucy C. Okell:** Writing – review & editing, Validation, Supervision, Methodology, Conceptualization. **Samson Kiware:** Writing – review & editing, Validation, Supervision, Methodology. **Gina Cuomo-Dannenburg:** Writing – review & editing, Validation, Methodology, Conceptualization. **Neterindwa Ainea:** Writing – review & editing, Validation, Supervision, Conceptualization. **Deus S. Ishengoma:** Writing – review & editing, Supervision, Conceptualization. **Nkuba Nyerere:** Writing – review & editing, Supervision, Methodology, Conceptualization.

## Ethical approval

Not applicable.

## Statement on the use of AI-assisted technologies

While preparing this article, the authors used OpenAI's to correct grammatical errors and improve readability. After using this tool, the authors reviewed and edited the content as needed. The authors take full responsibility for the content of the published article.

## Funding

This work was supported by the Gates Foundation [INV-070227 and INV-067322]. Under the grant conditions of the Foundation, a 10.13039/100026877Creative Commons Attribution 4.0 Generic License has already been assigned to the Author Accepted Manuscript version that might arise from this submission.

## Data availability

This study used data from the existing literature.

## Declaration of competing interest

The authors declare that they have no known competing financial interests or personal relationships that could have appeared to influence the work reported in this paper.
